# Cardiac Computed Tomography Identification of the Septal Vein—A Small Retrospective Study

**DOI:** 10.3390/life14060748

**Published:** 2024-06-12

**Authors:** Min Ku Chon, Ki Seok Choo, June Hong Kim

**Affiliations:** 1 Department of Cardiology, Pusan National University School of Medicine and Research Institute for Convergence of Biomedical Science and Technology, Pusan National University Yangsan Hospital, Beomeo-ri, Mulgeum-eup, Yangsan-si 626-770, Gyeongsangnam-do, Republic of Korea; chonmingu@gmail.com (M.K.C.); junehongk@gmail.com (J.H.K.); 2 Department of Radiology, Pusan National University School of Medicine and Research Institute for Convergence of Biomedical Science and Technology, Pusan National University Yangsan Hospital, Beomeo-ri, Mulgeum-eup, Yangsan-si 626-770, Gyeongsangnam-do, Republic of Korea

**Keywords:** heart, vein, CT, anatomy

## Abstract

Background: The advancement of medical interventions towards minimally invasive procedures highlights the crucial role of precise pre-procedural evaluation, particularly in catheter-based treatments for heart and cardiovascular conditions. This study investigates innovative techniques such as mitral loop cerclage (MLC) and transcatheter intramyocardial radiofrequency ablation (TIRA), emphasizing the importance of preprocedural cardiac CT scans for accurate anatomical guidance in these emerging therapies. Purpose: The objective of this study was to assess the cardiac cycle through examination of the proximal septal vein (ps) for mitral loop cerclage and the distal septal vein (ds) for transcatheter intramyocardial radiofrequency ablation. Materials and Methods: Forty patients (mean age 59.4 ± 14.7 years) undergoing third-generation dual-source computed tomography (DSCT) for chest pain evaluation were enrolled. CT scans, utilizing dual-energy CT (DECT) with iopamidol and saline, encompassed the carina to the heart base. A noise-optimized linear blended image was reconstructed at 10% intervals throughout the cardiac cycle, and the presence of ps and ds in each phase was noted by two radiologists. Results: This study identified ps in 62.5% and ds in 72.5% of patients, with both present in 45% of cases. The observation of septal veins occurred more frequently in the sequence of 70, 60, 40, 80, 30, 20, and 10% for ps, and 60, 70, 40, 80, 30, 90, 20, and 10% for ds, respectively. Conclusions: DECT in cardiac imaging is instrumental in assessing septal vein frequency. The 70% phase is optimal for MLC, while the 60% phase is preferred for TIRA.

## 1. Introduction

As modern medicine advances from traditional surgical treatment towards minimally invasive procedures, the importance of accurately evaluating a patient’s condition pre-procedure cannot be overstated. Specifically, catheter-based, minimally invasive treatments have emerged as crucial methods for combating heart and cardiovascular diseases. Medical practitioners undertaking these procedures must gather detailed information about blood vessels in the heart, including aspects such as their presence, shape, and size. To obtain this essential information, patients typically undergo a series of diagnostic tests like angiography, computed tomography (CT), magnetic resonance imaging (MRI), and chest radiography. These tests provide medical professionals with crucial medical images that are then utilized to design appropriate protocols and analysis methods for precise information acquisition and subsequent analysis.

Septal veins, also known as septal perforating veins, are vessels that commonly branch off from the anterior and inferior interventricular segments of the great cardiac vein. These veins typically emerge at right angles and navigate through the interventricular septum. Previous research by the authors has presented innovative treatments that involve penetrating the septum directly or targeting the ventricular septum. One such novel approach is the Mitral loop cerclage (MLC), a unique annuloplasty technique that leverages the coronary sinus (CS). This method offers an advantage in terms of accessibility and procedural simplicity compared to previously proposed catheter-based CS annuloplasty treatments [1,2,3]. During the MLC procedure, the basal septal veins are often chosen to target the basal part of the interventricular septum and then traverse to the right ventricle. A guidewire and cerclage rope follow this path to form a circular configuration around the mitral valve. Another technique, Transcatheter intramyocardial radiofrequency ablation (TIRA), is a localized treatment method developed for hypertrophic obstructive cardiomyopathy (HOCM) [4]. Similar to MLC, TIRA is a transvenous intraseptal radiofrequency ablation approach that passes through the CS to access the lesion site within the ventricular septum using the septal vein. Selecting a proximal septal vein that extends deeply into the ventricular septum is crucial for accurate target site access in this procedure. Additionally, septal para-hisian pacing has been proposed as an alternative treatment option employing a similar approach. Imaging guidance, particularly through preprocedural cardiac CT scans, plays a pivotal role in visualizing the anatomy of septal veins [5,6,7]. It serves as a key imaging tool for determining and planning the procedure in advance.

The primary objective of this study is to evaluate the cardiac cycle by examining the proximal septal vein (ps) for MLC and the distal septal vein (ds) for TIRA procedures, enhancing the understanding and precision of these minimally invasive cardiac interventions.

## 2. Materials and Methods

### 2.1. Study Population

This was a retrospective study approved by the Institutional Review Board of, which waived the requirement for informed consent. Between May and October 2021, 40 consecutive patients with chest pain who visited our hospital underwent dual-energy cardiac CT (Somatom Force, Siemens Healthineers, Forchheim, Germany), and were included in the study. The exclusion criteria were reduced renal function (estimated glomerular filtration rate < 45 mL/min per 1.73 m^2^) and previous adverse reactions to iodinated contrast agents. Based on these exclusion criteria, all 40 patients (21 men and 19 women, mean age 59.4 ± 14.7 years) were enrolled in the study.

### 2.2. CT Protocol

In this study, imaging data were meticulously acquired using a state-of-the-art third-generation dual-source CT (DSCT; Somatom Force, Siemens Healthineers, Forchheim, Germany). To facilitate coronary artery dilation, sublingual administration of nitroglycerin spray (0.4 mg, nitrolingual^®^, Mckesson Medical-Surgical, Inc.) was conducted 5 minutes prior to examination, while beta-blockers were intentionally withheld to maintain a mean heart rate of 68.0 ± 11.4 bpm. CT scans were performed subsequent to the continuous infusion of an 80 mL bolus of Ioversol (Optiary^®^, 320 mg/mL, Reyonpharm, Seoul, Republic of Korea), followed by a 30 mL saline infusion via an 18-gauge catheter inserted into the antecubital vein at an infusion rate of 5 mL/s. The bolus chase method was employed to evaluate both coronary arteries and veins. Image acquisition commenced after the placement of a region of interest at the root of the aorta, initiating 11 seconds after the signal attenuation reached a predefined threshold of 100 Hounsfield units (HU) to optimize contrast enhancement of the cardiac vein. Utilizing the retrospective scan mode in dual-energy mode (90 kVp and 150 kVp, gantry rotation time: 250 ms), scans were conducted from the level of the carina to the base of the heart in a craniocaudal direction, employing a detector collimation of 2 × 192 × 0.6 mm and a z-flying focal spot technique. To ensure optimal image quality, noise-optimized linearly blended images were automatically reconstructed with a blending factor of 0.6 (F_0.6), covering 60% of the low tube voltage (90 kVp) and 40% of the high tube voltage (150 kVp). Reconstruction parameters included model-based iterative reconstruction strength at level five (ADMIRE; Siemens Medical Solutions, Forchheim, Germany), utilizing a Bv40 medium smooth kernel and a section thickness of 0.75 mm in 0.3 mm increments. Acquired images were subsequently reconstructed at 10–100% of the R-R interval in 10% increments to facilitate the assessment of the proximal septal vein (ps) and distal septal vein (ds), enabling comprehensive evaluation across various phases of the cardiac cycle.

### 2.3. Image Analysis

Following acquisition, all image datasets were securely transferred to a specialized workstation (AquarisNet Viewer 1.8.0.3; TeraRecon, Foster City, CA, USA) and a robust picture-archiving communication system (Maroview 5.4; Infinitt, Seoul, Republic of Korea) for comprehensive analysis. Utilizing the dedicated workstation, axial images spanning the entire cardiac cycle (0–100%) were reconstructed to assess the proximal septal vein (ps), while short-axis images were generated to evaluate the distal septal vein (ds). Subsequently, a rigorous assessment of the images was conducted by a multidisciplinary team consisting of a seasoned radiologist with 20 years of experience and a cardiologist with 15 years of expertise. Each expert meticulously analyzed the visibility of ps and ds across various phases of the cardiac cycle, ensuring comprehensive scrutiny and precise identification. Ps was defined as a vein heading toward the right ventricular outlet (RVOT), and ds was defined as a vein heading toward the central ventricular septum. To maintain consistency and reliability, all analyses were performed under strict agreement between the two experts, minimizing interobserver variability and enhancing the robustness of the findings.

## 3. Results

**Figure 1:**
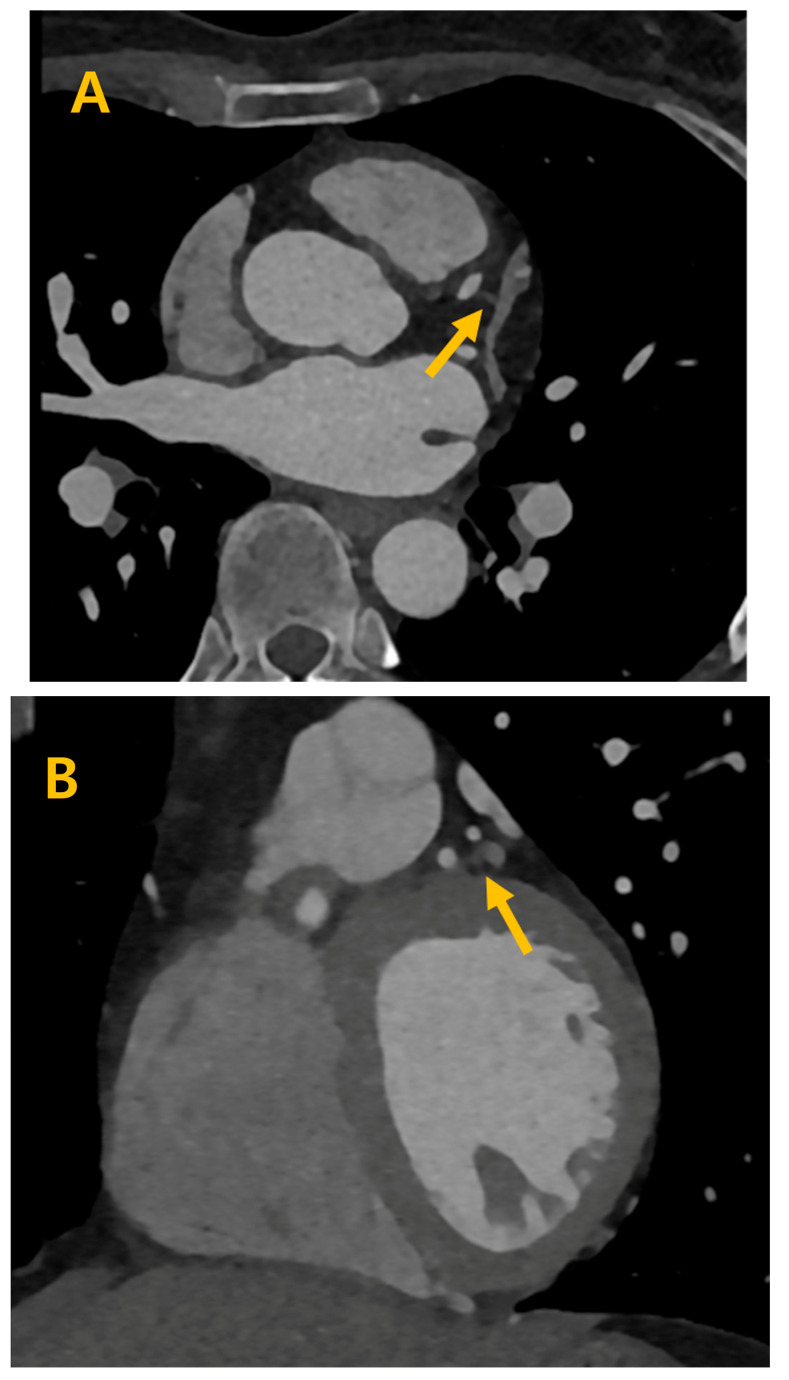

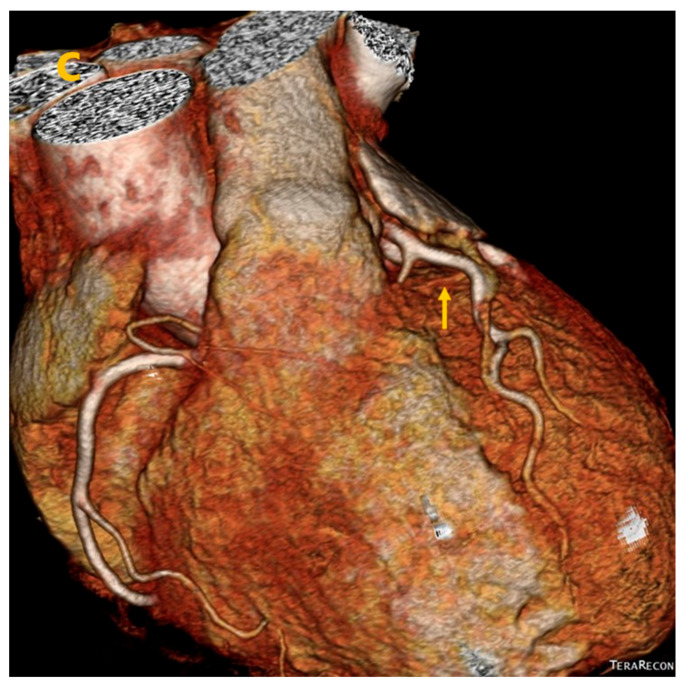
Proximal septal vein (ps) is distinctly discernible, clearly indicated by the arrow, in both the axial (**A**), short-axis (**B**) and 3 dimensional views (**C**) during the 70% cardiac phase, demonstrating its prominent visibility and anatomical significance.

**Figure 2:**
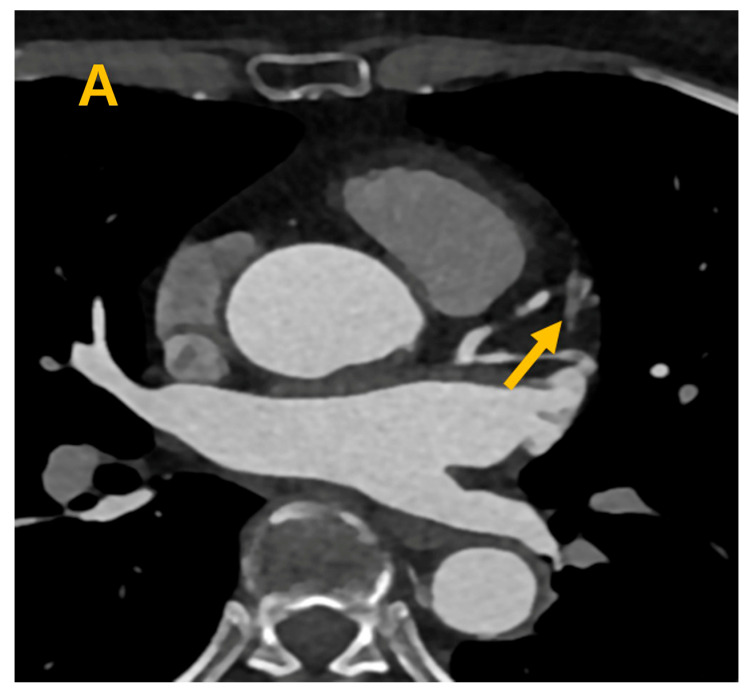

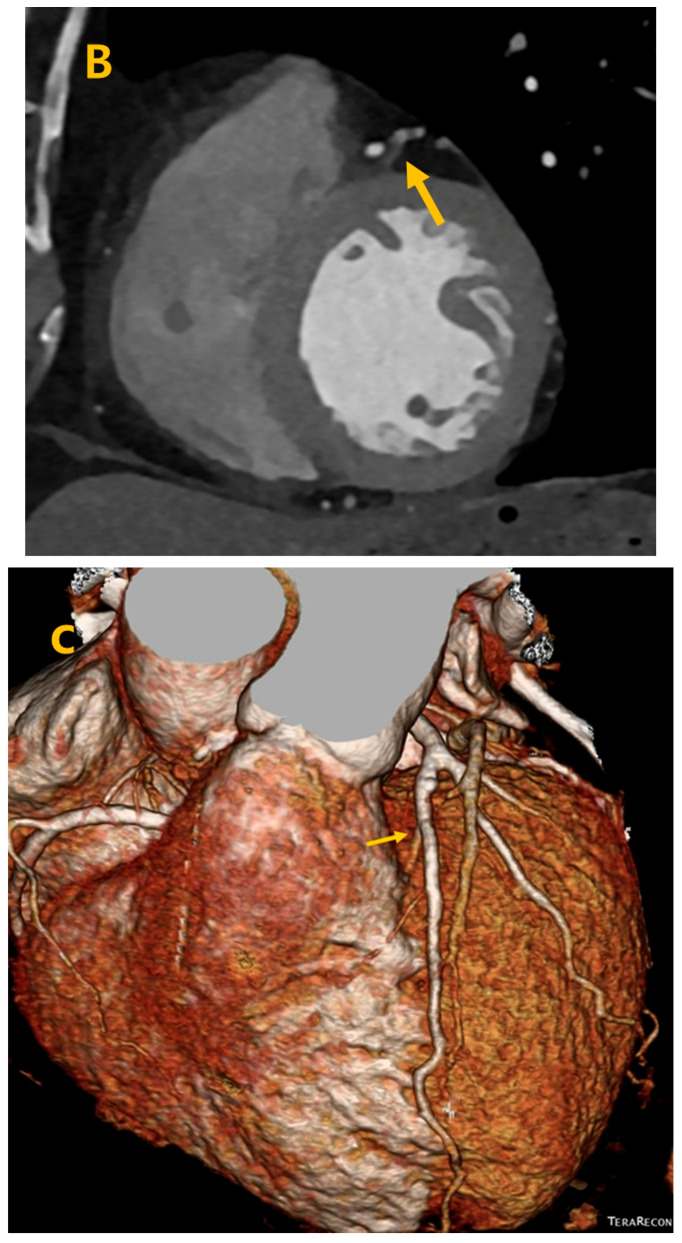
During the 60% cardiac phase, the distal septal vein (ds) is prominently depicted, highlighted by an arrow, in both the axial (**A**), short-axis (**B**) views and three-dimensional views (**C**), emphasizing its discernible presence and anatomical location.

Among the cohort of 40 patients studied, the proximal septal vein (ps) was identified in 25 patients, accounting for 62.5% of the total sample size (Figure 1). Similarly, the distal septal vein (ds) was visualized in 29 patients, representing 72.5% of the study group (Figure 2). Notably, both the ps and ds were concurrently observed in 18 patients, indicating a prevalence rate of 45%.

When looking at how often certain veins appear in patients, we found that septal veins were seen at different rates. In patients with ps, septal veins were most common at 70%, followed by 60%, 40%, and so on. In patients with ds, the order was slightly different, with the most common septal vein at 60%, followed by 70%, 40%, and so on (Table 1).

## 4. Discussion

This investigation was undertaken with the primary aim of identifying the most advantageous cardiac phase for conducting cardiac CT scans employing DECT to assess the proximal septal vein (ps) and distal septal vein (ds) before MLC and TIRA procedures. Among the various cardiac phases scrutinized, the study determined that the 70% cardiac phase was optimal for MLC, while the 60% phase was deemed ideal for TIRA. Although the focal point of this study revolved around MLC and TIRA procedures, it is imperative to acknowledge the broader clinical relevance of cardiac veins across diverse surgical and procedural contexts beyond MLC and TIRA. Cardiac veins frequently serve as access points for electrophysiological interventions such as cardiac resynchronization therapy and implantable cardioverter-defibrillator implantation, involving the placement of electrodes or devices within the heart to enhance electrical function and manage arrhythmias. There are different ways to take pictures of the coronary veins, each with its own good and bad points. For instance, with the improvements in coronary artery MRI, we can now use MRI to see the coronary veins. MRI is helpful because it shows the shape, how well the heart is working, and if there is any damage, all in one test. But it takes a long time and costs a lot compared to another method called DECT. The incorporation of the F_0.6 algorithm using dual-source DECT, as advocated by Kim et al. [8], yielded superior signal-to-noise ratio (SNR) and contrast-to-noise ratio (CNR) compared to other reconstruction algorithms. Embracing this protocol and algorithm in our study addressed the challenges posed by the relatively low enhancement and high noise associated with cardiac vein imaging compared to arteries, thereby mitigating some inherent limitations in examining cardiac veins. The precise identification and evaluation of the ds and ps of septal veins via CT scans play a pivotal role in confirming the feasibility of minimally invasive procedures while potentially reducing procedural duration by pinpointing the exact location of septal veins. However, it is crucial to acknowledge that not all patients may possess suitable septal veins, as some may be too small or not fully developed to be visualized through imaging. After the procedure, regular follow-up DECT allows for monitoring the patient’s progress, assessing the effectiveness of the procedures, and detecting any potential complications or issues that may arise over time. This ongoing observation is crucial for understanding the long-term benefits of the procedures and ensuring the patient’s overall health and well-being. This study acknowledges certain limitations. Firstly, this study has a small sample size and is retrospective in nature. In the future, multi-center trials or studies incorporating a wider variety of cardiac conditions will be needed to validate and expand upon the findings. Secondly, the scope of treatments based on CT imaging analysis of septal veins remains somewhat constrained. Innovations such as cerclage mitral regurgitation treatment, HOCM ablation, and septal para-hisian pacing, spearheaded by the research team, are still in their nascent stages and require further comprehensive investigations to enhance precision and applicability. Finally, challenges persist in identifying septal veins and their branches during live procedures, particularly when they may have been overlooked during CT analysis due to factors such as fine vasculature that elude current imaging resolutions and are influenced by patient factors like respiration and cardiac movements. Developing protocols to enhance visibility and accuracy through subsequent studies is imperative. In conclusion, cardiac CT utilizing DECT emerges as a valuable tool for assessing septal veins, with the 70% phase identified as optimal for MLC and the 60% phase for TIRA. Continued advancements in technology and research are crucial to improving the accuracy and utility of septal vein analysis for a range of cardiovascular procedures.

## Figures and Tables

**Table 1:** Comparing 10–100% of cardiac cycle phases for assessment of proximal septal vein (ps) and distal septal vein (ds).

Order of Prevalence for Ps	Order of Prevalence for Ds
70% (20/25)	60% (22/29)
60% (17/25)	70% (20/29)
40% (12/25)	40% (17/29)
80% (9/25)	80% (7/29)
30% (6/25)	30% (6/29)
20% (3/25)	90% (3/29)
10% (2/25)	20% (1/29)
	10% (1/29)

## Data Availability

Data is unavailable due to privacy or ethical restrictions.
